# Using a quality improvement process to improve cataract outcomes

**Published:** 2022-12-16

**Authors:** Chandrashekharan Shivkumar, Haripriya Aravind, Ravindran D Ravilla

**Affiliations:** 1 Cataract and IOL services: Aravind Eye Hospital, Tirunelveli, India.; 2 Chief: Cataract and IOL services, Aravind Eye Hospital, Chennai, India.; 3 Chair: Aravind Eye Care System, Madurai, India.

**Ongoing monitoring and a systematic approach to quality improvement can improve outcomes for patients.**

The Aravind Eye Care System (AECS) has an annual output of over 300,000 cataract operations a year through its network of 14 hospitals. More than 60% of all operations are subsidised or at no cost to the patient, and they are performed using the manual small-incision cataract surgery (MSICS) technique.

Thanks to advances in surgical techniques and intraocular lens (IOL) technology, cataract surgery can now restore sight and address refractive error. Given that many patients may not have access to spectacles, or be able to afford them, it is important to achieve a good presenting visual acuity after surgery. In recognition of recent evidence about the impact of mild vision impairment (visual acuity of <6/12 to 6/18) on the everyday functioning of individuals,^1,2^ the World Health Organization now recommends a threshold for presenting visual acuity after cataract surgery of 6/12 or better.^3^

As part of Aravind’s ongoing cataract quality improvement strategy, we set out to address postoperative presenting visual acuity by testing a different approach to biometry. Biometry is the process of taking measurements of the eye to predict the power of IOL that would be needed by each patient. Accurate prediction of IOL power is one of the major factors that determines presenting visual acuity after cataract surgery. The accuracy of a biometry service is measured by recording the percentage of patients for whom the difference between the target refraction (estimated during biometry) and the refraction achieved after surgery falls within a specified range of prediction error; this is expressed as a spherical equivalent, in dioptres (D).

Our quality improvement process includes these steps:

1. Identify the problem (ask: what needs to change?) and gather baseline data on outcomes/outputs before changes are made
2. Set standards based on agreed benchmarks
3. Decide on the methods or equipment needed to make an improvement
4. Introduce changes and train personnel
5. Measure impact
6. Gather data to drive a process of ongoing improvement.

## Identifying the problem and gathering baseline data

**Figure 1:**
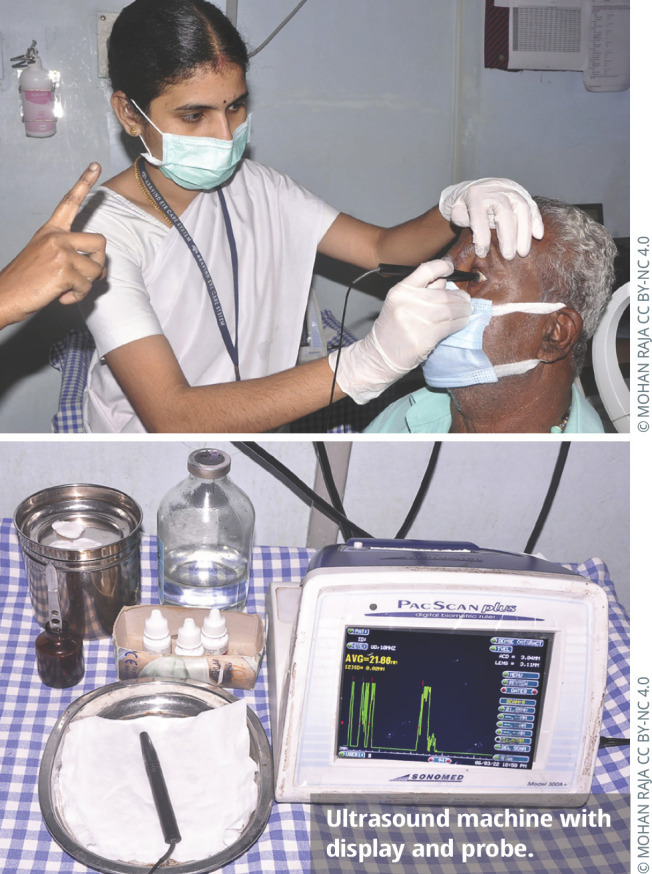
A biometrist performs contact (applanation) ultrasound biometry. **INDIA**

Until 2012, IOL power was calculated using contact or applanation ultrasound biometry methods as this is easy and quick to perform, especially in high-volume services (Figure 1). However, because this method involves direct contact with the cornea, compression of the cornea is possible, which can cause reading errors.

Aravind uses an electronic medical record-keeping system called CatQA to monitor and continually improve outcomes and processes. When we analysed the CatQA data from our hospitals, we found that just 40.4% of the patients who had undergone ultrasound biometry and MSICS had a prediction error within ± 0.5D, and 85% had a prediction error within 1.0D.

## Setting standards

We decided to base our standards for the accuracy of biometry on the benchmark set by the UK’s National Health Service (NHS): a prediction error within ±0.5 D in 60% of patients, and within ±1.0D in 90% of patients.^4^

## Finding the methods or equipment needed to make an improvement

There is good evidence^5^ that immersion ultrasound biometry performs better than contact ultrasound biometry and can be used in all cataract types (although optical biometry performs better than ultrasound overall, it doesn’t work in advanced cataract – which is more typical in low-income settings such as ours).

Based on this evidence, and our available human and financial resources, we took the decision to convert from applanation ultrasound biometry to immersion ultrasound biometry in all 14 eye hospitals.

## Introducing changes gradually

**Figure 2:**
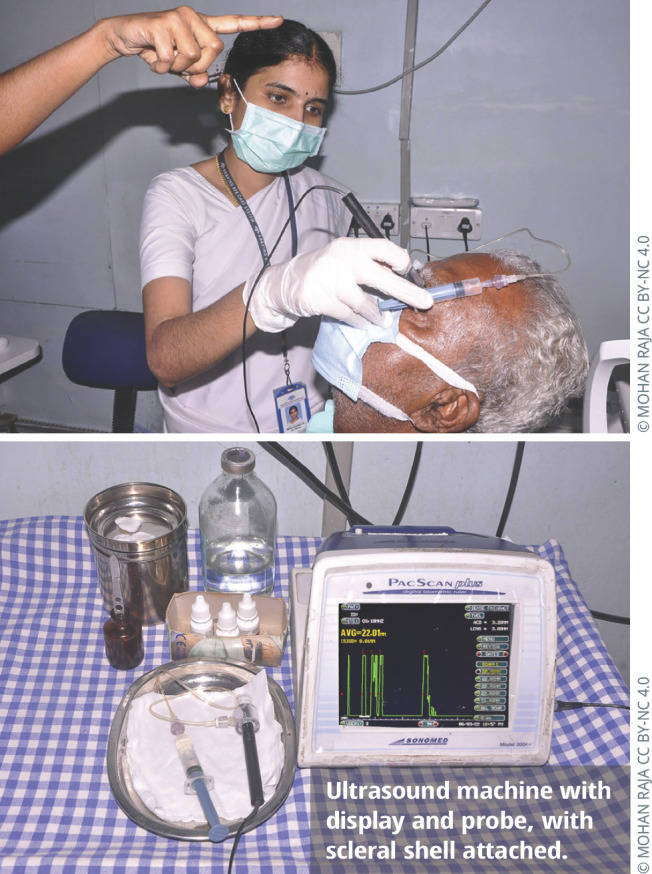
A biometrist performs immersion ultrasound biometry. **INDIA**

Immersion biometry was implemented between 2013 and 2018, in just a few hospitals at a time, by first upgrading the equipment and then retraining the staff members who perform biometry. Training was structured and staff were closely monitored. By the end of 2018, al 14 hospitals were performing immersion ultrasound biometry (Figure 2).

## Measuring impact

To measure impact, we collected data about the accuracy of IOL power prediction a year after introducing immersion ultrasound biometry and again in 2021. The impact of the change was evident when we compared this with the baseline data from 2012 (Table 1).

**Table 1:** Patients seen in a 12-month period with prediction error within ± 0.5D and 1.0D (the benchmarks are 60% within 0.5D and 90% within 1.0D)

	Prediction error within ± 0.5D	Prediction error within ± 1.0 D
**Contact biometry (2012)**	46,278 (40.4 %)	97,410 (85.0 %)
**Immersion biometry 1 year after it was introduced**	84,036 (54.6 %)	147,758 (96.0 %)
**Immersion biometry (March 2022)**	71,871 (67.7%)	101,874 (96.0%)

Following the adoption of immersion ultrasound procedure across all 14 hospitals, we found that, of the 153,868 patients who had undergone immersion biometry, 54.6% now had a prediction error within ± 0.5D (up from 40.4%) and 96.0% had a prediction an error within ±1.0D (up from 85%).

## Ongoing data gathering and evaluation

**Figure 3:**
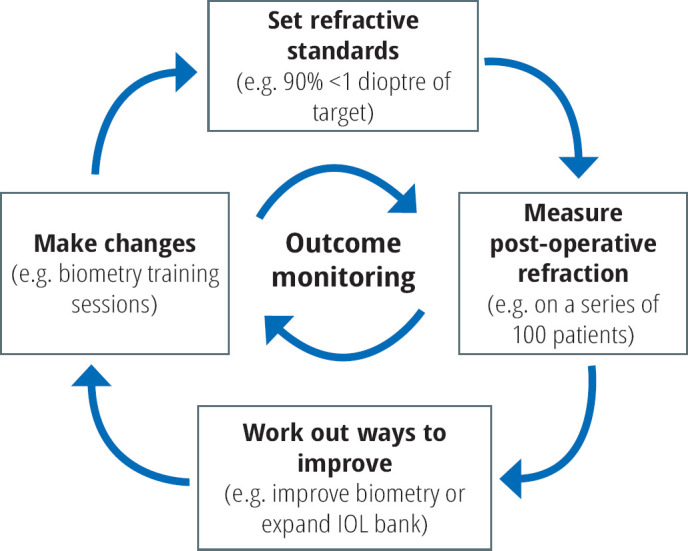
Continuous monitoring of outcomes to improve quality

We continued to routinely monitor the prediction error and make improvements where needed. (Figure 3), using a process of outcome monitoring and quality improvement.

Other opportunities for quality improvement, including using better IOL calculation formulae and offering staff members further biometry training, were responsible for some of the additional improvements seen between 2019 and 2021 (Table 1).

In 2021, despite carrying out fewer operations, on more advanced cataracts (due to the COVID-19 pandemic), we significantly exceeded the NHS benchmarks with 68.2% and 94.9% of patients within the ±0.5 D and ±1.0D prediction error, respectively (Table 1).

There was a corresponding improvement in the proportion of patients achieving better postoperative visual acuity once we started using immersion biometry. The proportion of patients who had uncorrected postoperative visual acuity of 6/18 or better improved from 63.0% in 2012 to 83.9% in 2021 (Table 2). Similarly, the proportion of patients with uncorrected visual acuity of 6/12 and better increased from 31.0% in 2012 to 59.8% in 2021 (Table 2).

**Table 2:** Patients seen in a 12-month period with uncorrected visual acuity (UCVA) of 6/18 or better and 6/12 or better, before and after adopting the immersion biometry technique.

	Number (and percentage) of patients achieving UCVA of 6/18 or better	Number (and percentage) of patients achieving UCVA of 6/12 or better
**Contact biometry** **(2012)**	114,560 (63.0%)	34,936 (31.0%)
**Immersion biometry** **(2021)**	89,560 (84.4%)	61,587 (58.0%)

To conclude, this process of patient-centred quality improvement promoted patient safety, treatment effectiveness, and efficient use of resources. The constant monitoring of outcomes provided the information necessary to continuously improve, refining the quality processes in ways that were often not expensive (e.g., using better IOL calculation formulae). The first step in the process is identifying where opportunities exist to improve, which will be different for each institution.

We would encourage everyone involved in cataract surgical service provision to be in this constant quality improvement cycle, as this helps to achieve the best outcomes for patients, irrespective of the volume of cataract surgery.

## Acknowledgements

*We acknowledge the contributions of Mr Thulasiraj Ravilla, Executive Director and Ms Dhivya Ramsamy, Senior Faculty of Lions Aravind Institute of Community Ophthalmology (LAICO), AECS Madurai*.
